# Draft Genome Sequence of Exophiala mesophila, a Black Yeast with High Bioremediation Potential

**DOI:** 10.1128/genomeA.00203-15

**Published:** 2015-04-09

**Authors:** Hakim Tafer, Ksenija Lopandic, Barbara Blasi, Caroline Poyntner, Katja Sterflinger

**Affiliations:** VIBT-Extremophile Center, University of Natural Resources and Life Sciences, Vienna, Austria

## Abstract

The fungal genus *Exophiala* comprises both pathogen species, which cause severe infections in humans, and environmental species, which are able to degrade alkylbenzene compounds. The draft genome sequence of Exophiala mesophila presented here is the first genome assembly of an alkylbenzene-degrading organism belonging to the genus *Exophiala*.

## GENOME ANNOUNCEMENT

So-called black yeasts are ascomycetes exhibiting hyphal and yeast-like morphology with budding cells. Within the black yeasts, the genus *Exophiala* seems to be an evolutionary hot spot, with a high diversification and emerging adaptation toward human environments and human hosts. Exophiala dermatitidis, in particular, is both an opportunistic human pathogen and a fungus found in human environments, like bathrooms, sauna facilities, or dishwashers. Some species show a high affinity for contaminated sites and are able to degrade a variety of xenobiotics, including hydrocarbons, under desiccation and under extreme pH and temperature conditions. Thus, they are regarded as promising candidates for bioremediation (1). Exophiala mesophila was originally isolated from a silicone seal in a hospital but is also frequently found in tap water, saunas, and steam bath facilities (2).

Genomic DNA was isolated from E. mesophila (strain CBS 120910) grown on 2% malt extract agar using a cetyltrimethylammonium bromide (CTAB)-based protocol. The elimination of melanin from DNA was performed by two phenol-chloroform purification steps. Genome sequencing was carried out using the Ion Proton Technology (Ion AmpliSeq library preparation kit, Template OT2 200 kit, Ion PI sequencing 200 kit, and the Ion PI chip kit version 2; Life Technologies, Carlsbad, CA), according to the instructions of the producers. A total of 12.2 Gb, with a median read length of 169 bp, were assembled into a 30-Mb genome containing 507 contigs (*N*_50_, 121,790). The genome assembly was performed with Newbler 2.9.

The presence of genes involved in toluene degradation was investigated in E. mesophila and, for the purposes of comparison, was also investigated in the yeast Saccharomyces cerevisiae S288C. A list of 12 genes related to toluene degradation was compiled from Parales et al. (3) and mapped to the three genomes using Scipio (4), with a score threshold of 0.3. Only 2 genes belonging to the toluene degradation pathway (benzaldehyde dehydrogenase and benzylalcohol dehydrogenase) were found in yeast S288C, while 9 genes (ketoadipyl-coenzyme A [CoA] thiolase, catechol 1,2-dioxygenase, protocatechuate dioxygenase, cytochrome P450, benzaldehyde dehydrogenase, benzylalcohol dehydrogenase, *cis*,*cis*-muconate-lactonizing enzyme, β-carboxy muconate enzyme, and β-ketoadipate-CoA transferase) were found in E. mesophila, in line with its ability to digest alkylbenzene compounds.

### Nucleotide sequence accession numbers.

This whole-genome shotgun project has been deposited at DDBJ/EMBL/GenBank under the accession no. JSEI00000000. The version described in this paper is JSEI01000000.
